# The creation of phenomena in interactive biorobotics

**DOI:** 10.1007/s00422-021-00900-x

**Published:** 2021-10-29

**Authors:** Edoardo Datteri

**Affiliations:** RobotiCSS Lab, Laboratory of Robotics for the Cognitive and Social Sciences, Department of Human Sciences for Education, University of Milano-Bicocca, Milano, Italy

**Keywords:** Interactive biorobotics, Social behaviour, Explanation, Understanding

## Abstract

In so-called interactive biorobotics, robotic models of living systems interact with animals in controlled experimental settings. By observing how the focal animal reacts to the stimuli delivered by the robot, one tests hypotheses concerning the determinants of animal behaviour in social contexts. Building on previous methodological reconstructions of interactive biorobotics, this article reflects on the claim, made by several authors in the field, that this strategy may enable one to explain social phenomena in animals. The answer offered here will be negative: interactive biorobotics does not contribute to the explanation of social phenomena. However, it may greatly contribute to the study of animal behaviour by *creating* social phenomena in the sense discussed by Ian Hacking, i.e. by precisely defining new phenomena to be explained. It will be also suggested that interactive biorobotics can be combined with more classical robot-based approaches to the study of living systems, leading to a so-called simulation-interactive strategy for the mechanistic explanation of social behaviour in animals.

## Introduction

Robots have been used as models of living systems since the first decades of the XX century (Cordeschi 2002). In cybernetics and classical biorobotics, robotic models are experimentally deployed to explain and predict the behaviour of animals, human beings included (Gravish and Lauder 2018; Webb 2001; Webb and Consi 2001). The claim that robots may constitute experimental tools to study the behaviour and cognition of living systems has been recently revitalized in an emerging strand of biorobotics called ethorobotics (Romano et al. 2019a, b) or interactive biorobotics (Datteri 2020a). Interactive biorobotics—IB from now on—is distinctively characterized by experiments in which robots and animals interact in controlled settings (whereas no such interaction occurs in classical biorobotics). The robot delivers controlled social stimuli to the focal animal: its appearance and behaviour are manipulated, and the reactions of the animal are observed and analysed. The strategy of establishing such a “hybrid” social setting, involving robots and animals in mixed societies, is regarded by some scholars as a promising approach to study social behaviour in animals. Faria and colleagues (2010) claim that IB is “a novel method for studying collective animal behaviour” while, according to Krause and colleagues (2011), “interactive robots have the potential to revolutionise the study of social behaviour”. IB has been applied to the study of social behaviour of fish (Polverino et al. 2013), birds (Jolly et al. 2016; Patricelli and Krakauer 2010), dogs (Gergely et al. 2015), locusts (Romano et al. 2019, 2019b). Other studies will be cited in the following sections. For a comprehensive and updated review, see (Romano et al. 2019a, b).

Building on previous methodological reconstructions of the field (Datteri 2020a, 2021), this article offers a methodological reflection on the role that IB experiments play in the study of collective animal behaviour from the point of view of the philosophy of science. The goal is to reflect about a rather ambitious proposition set forth by many interactive biorobotics, stating that interactive robots can be used to *understand* social behaviour (Mitri et al. 2013). Phamduy and colleagues (2014) claim that “the experimental integration of bioinspired robots in groups of social animals has become a valuable tool to understand the basis of social behaviour and uncover the fundamental determinants of animal communication”. Bonnet and colleagues (2019) write that “recently, robots have been developed to collaborate with animal groups in the pursuit of better understanding their decision-making processes”. According to Fernández-Juricic and colleagues (2006) “robots have a great potential for advancing our understanding of the dynamics of social behaviour”. In the review made by Romano and colleagues (Romano et al. 2019a, b) it is claimed that “robots can be used to manipulate groups of living organisms to understand self-organization and the evolution of cooperative behaviour and communication”. Griparić and colleagues (2017) present “a novel robotic system developed for researching collective social mechanisms in a biohybrid society of robots and honeybees”, with the purpose of “understanding the principles of collective behaviour in social animals”. The term “understand” recurs in these statements, notwithstanding some differences in the object of the understanding (e.g. processes, dynamics, principles, behaviour). They all express the opinion that IB may advance our understanding of social phenomena in animals. Can IB experiments live up to this ambitious expectation?

One may be more or less inclined to endorse this thesis depending on the meaning assigned to “understand”. Philosophers of science have offered several analyses of the concept of “understanding” and of the relationship between understanding and explanation (Barnes 2010; de Regt 2009; Friedman 1974; Hu 2021; Trout 2002). Michael Strevens (2013), in particular, argues that understanding without explanation is impossible: one has a scientific understanding of a phenomenon just in case they grasp a good explanation of that phenomenon. According to this construal of the concept of understanding, IB experiments may advance our understanding of social phenomena in animals only if they result in the formulation or testing of good explanations of social phenomena. Note that classical—non-interactive—biorobotics has been regarded, by philosophers of science, as a strategy to formulate and test (mechanistic) explanations of the behaviour of living systems (Darden 2017; Datteri and Tamburrini 2007). Can IB play the same epistemic role in the study of animal behaviour? Are IB experiments carried out to formulate or test explanations of social behaviour in animals? This is the specific question addressed in this article.

The answer offered here will be negative: the goal of IB studies is not to explain social phenomena. Nor they end up formulating or testing explanations of social phenomena. If one endorses the “no understanding without explanation” thesis defended in (Strevens, 2013), IB experiments, per se, do not advance our understanding of social phenomena. This by no means undermines the thesis that IB constitutes an interesting approach to the study of social behaviour in animals. Indeed, it will be claimed that IB experiments end up *creating* social phenomena in the sense famously discussed by Ian Hacking in his *Representing and Intervening* (1983): their distinctive epistemic role is to define the contours of social phenomena which await explanation. Creating new phenomena is an important contribution to the study of animal behaviour, and it is in this sense that interactive biorobotics has the potential to revolutionise the study of social behaviour as claimed by Krause and colleagues (2011).

The article is structured as follows. Building on previous methodological analyses, the methodological structure of IB is discussed in Sect. 2, by comparing it with classical biorobotics (2.1), reconstructing the structure of the theoretical conclusions reached in IB studies (2.2), and analysing the structure of the research questions motivating IB studies (2.3). These sections will lay the foundations for Sect. 3, which reflects on whether IB studies may offer explanations of social phenomena in animals. Section 4 offers some concluding remarks and suggests possible combinations of interactive and classical biorobotics for the explanation of the phenomena created in IB studies, outlining a so-called simulation-interactive methodology.

## Interactive biorobotics

### Models: from surrogative reasoning to stimulation

The distinctive characteristics of interactive biorobotics can be best appreciated by comparing it with the methods of classical, non-interactive biorobotics, which have been applied to the study of bats (Bou Mansour et al. 2019), crickets (Reeve et al. 2005), desert ants (Lambrinos et al. 2000), lobsters (Grasso et al. 2000), locusts (Blanchard et al. 2000) and many other living systems, human brains included (for reviews, see Datteri 2017; Gravish and Lauder 2018; Webb 2002, 2001; Webb and Consi 2001). Classical biorobotics is based on the so-called synthetic method (Cordeschi 2002; Pfeifer et al. 2008). The goal is to discover the mechanism M governing some aspect of the behaviour of target living system T (where “T” stands for “target”). A robot R is built which implements mechanism M. If the robot R succeeds in reproducing T’s behaviour, one may be induced to corroborate the hypothesis that M is the mechanism governing T’s behaviour too. Otherwise, one may be induced to exclude M from the space of the possible explanatory mechanisms. This strategy is called “classical” here because it has been adopted since the first decades of the XX century (Cordeschi 2002), and because it is methodologically akin to a variety of model-based studies carried out in other research fields including physics, biology, economics, where (non-robotic) models are used to discover and test mechanistic models of target systems. Classical biorobotics instantiates what has been called *surrogative reasoning* (Frigg and Nguyen 2017; Swoyer 1991): the robotic model R is a surrogate on which one can perform experiments to learn something about the target system T.

Classical biorobotics significantly differs from interactive biorobotics in a number of methodological respects. One of the most striking differences is that the former does not involve any interaction between robots and real-life biological systems, while interaction between the robot and the living system under investigation is crucial in the IB methodology. In IB one builds a robot R which may (but needs not) model some characteristics of a living system T. For example, the robot built by Romano and colleagues and described in (Romano et al. 2019, 2019b) modelled some characteristics of a gecko. However, while in classical biorobotics T—the modelled living system—is the system under investigation, about which the experimenter draws theoretical conclusions based on the analysis of surrogate R, in interactive biorobotics the system under investigation is *not* the modelled system T. The robot is not used as a surrogate to reason on T: it is rather used to stimulate *another living system*, which will be called here *focal* system (F). More succinctly, in classical biorobotics the robot R models living system T and is used to theorize on T, while in interactive biorobotics the robot R models living system T and is used to theorize on *another* living system F. R stimulates the focal system F by interacting with it in controlled experimental settings, and the characteristics of R can be selectively manipulated so that it delivers specific stimuli to F. The experimenter analyses the reactions of the focal system F to the stimuli delivered by the robotic model. For example, Romano and colleagues (Romano et al. 2019a, 2019b) analysed the motor reactions of a locust (the focal system F) to the stimuli delivered by a robot R modelling a gecko (T). Note that in IB the system under investigation is part of the experimental scenario, contrary to classical biorobotics, in which the system under investigation is experimentally replaced by its robotic surrogate. The distinction between classical and interactive biorobotics will not be further analysed here, as it is has been thoroughly discussed in (Datteri 2020a, 2021).

### Theoretical conclusions in interactive biorobotics

What can be learned about focal system F by observing how it reacts to robot R? Datteri (2020a) distinguishes between *proximal* and *distal* IB studies. The goal of proximal studies is to find out how the focal system reacts to the robotic model whenever it displays certain features. For example, Polverino and colleagues (2013) studied the interaction between a robotic fish and real-life fish in a water tunnel. They measured the distance between the robot and the focal fish in conditions differing from one another in the characteristics of R and found out that, whenever the robotic fish had a realistic pigmentation and a certain tail-beat frequency, the focal fish tended to approach the robot. Let us use label P_R_ to refer to a description of the manipulated characteristics of the robot and P_F_ to refer to a description of the characteristics of the focal system under investigation. Proximal studies end up establishing relationships stating that, whenever the robot displays characteristics P_R_, the focal system will display characteristics P_F_. Schematically,

$$ \left( {{\text{prox}}} \right) \, P_{R} \to P_{F} $$

Assessing how a living system reacts to a robot may constitute an interesting result per se, with important technological implications and potential applications (e.g. for the selective “rewiring” of ecosystems as in Bonnet et al. 2019). So-called *distal* IB studies push the approach one step further. In a distal study, one assesses how the focal system F reacts to the robot R in order to infer how the focal system F would react to the living system T modelled by the robot. For example, Butler and Fernández-Juricic (2014) observed how a real-life starling (F) reacted to a robotic starling (R) having characteristics P_R_ in order to infer how F would react to the real-life starling T modelled by R. More succinctly, in distal studies, a conclusion of the form P_R_ → P_F_ is used as a premise to infer a conclusion of the form

$$ \left( {{\text{dist}}} \right) \, P_{T} \to P_{F} $$

where P_T_ stands for a description of the characteristics of the living system modelled by the robot. *Dist* can be inferred from *prox* only assuming auxiliary assumptions concerning the relationship between R and T, which will not be discussed here (see Datteri 2021).

One limitation of the analysis carried out in (Datteri 2020a) is that it is based on case studies involving interactions between *individual* robots and *individual* focal systems only. However, IB studies may establish one-to-one, one-to-many, many-to-one, and many-to-many forms of interaction among robots and living systems.

A case of *one-to-one interaction* between an individual robot and an individual living system is described in (Butler and Fernández-Juricic 2014), which will be referred to, from now on, as “the starling study”. It concerns gaze following in starlings**.** Gaze following is the ability of orienting one’s own gaze towards the locus of visual attention of another individual. The authors of the starling study experimentally manipulated gaze direction (P_Ri_, where “i” stands for “individual”) of an individual starling-like robot and measured the gaze direction (P_Fi_) of a real-life starling located nearby. At the end of the study, they gathered evidence supporting the hypothesis that, whenever the robotic starling gazes at some point in space (an empty container in the experimental arena), the real-life starling will tend to gaze at the same point. Schematically,

$$ P_{{{\text{Ri}}}} \to P_{{{\text{Fi}}}} . $$

In other studies, *one* robot interacts with *many* living systems. The goal in that case is to establish a relationship between the characteristics P_Ri_ of an individual robot and the characteristics P_Fc_ (where “c” stands for “collective”) of a group of living systems. In (Michelsen et al. 1992), which will be called “the bee study” from now on, a mechanical model is described which is able to reproduce the dance that honeybees make to inform their nestmates of the distance and direction of food sources. The dance has several components, which include segments in which the bees rapidly wag, emitting sound patterns. The authors could manipulate some components of the dance produced by the model—e.g. include more wagging runs, increase or decrease the length of the wagging runs, increase or decrease sound emission time. The goal was to find out what dance components signalled position and distance of the food source. To this end the authors monitored the number of bees approaching food sources located at various distances and directions from the mechanical model, while the latter performed the “normal” or modified forms of the dance. At the end of the study, they found out that modifying some aspects of the mechanically produced dance made the difference, as different dances resulted in different patterns of aggregation of the bees at the various food sources. The study therefore established a regular relationship between the characteristics of an individual robot and the (behavioural) characteristics of a group of focal systems: schematically,

$$ P_{{{\text{Ri}}}} \to P_{{{\text{Fc}}}} . $$

Other IB studies involve interactions among *many* robots and *one* focal system: the study described in (Reaney 2009), called here “the crab study”, is a case in point. The question addressed in the study concerns the criteria used by female crabs to select their mates. Claw size and claw wave rate are known to be important factors. Very often, female crabs simultaneously confront with several males differing both in claw size and wave rate. Do they choose by attributing an absolute preference value to each male (possibly by combining evaluations of its claw size and wave rate), or by performing comparative evaluation, in which the attractiveness of each male depends comparatively on the attractiveness of the males nearby? Suppose that male A has a very attractive claw size and a non-attractive wave rate, while male B has a non-attractive claw size and a very attractive wave rate. A is more attractive than B in one important factor (claw size), while B is more attractive than A in the other factor (wave rate). If the two factors have equal weight, the female should have no reason to prefer A to B. Now suppose that a third male, C, is added such that A is more attractive than C in both factors, and B is more attractive than C in one of the two factors only. If the female used an absolute evaluation system, the preference for A or B should not change. Instead, if the female used a comparative evaluation system, the addition of C should modify the attractiveness of A relative to B (one might expect that the female will be more attracted to A, which is now superior to C along the two dimensions). The author of the crab study performed this test in experiments in which A, B, and C were robots, and the female was a real-life crab. The goal was therefore to establish correlations between the characteristics P_Rc_ of a group of robots and the behaviour P_Fi_ of one focal system: for example, to assess whether, in the presence of A, B, and C having the characteristics illustrated before, the female would prefer A. Schematically, the study aimed at establishing a regular relationship of the form

$$ P_{{{\text{Rc}}}} \to P_{{{\text{Fi}}}} . $$

Finally, some IB studies establish interactions among *many* robots and *many* focal systems. A case in point is the study reported in (Halloy et al. 2007), which will be called “the cockroach study” from now on. The subject of inquiry is the collective behaviour of cockroaches. When put in an arena with two dark shelters, cockroaches will eventually gather under one of them, after carrying out what can be properly described as a collective decision-making process. If one shelter is darker than the other one, they will approach it. The authors of the study replaced a few insects with an equal number of InsBots, i.e. little mobile robots more or less of the same size of a cockroach and coated with their characteristic odour. The InsBots were able to casually wander in the arena, avoid obstacles, recognize and avoid real-life cockroaches. When encountering a shelter, they waited there for a time depending on the darkness of the shelter and on the number of individual staying there (they preferred darker and more crowded shelters). A first result of the study was that, when InsBots displaying these characteristics were inserted into the group of real-life cockroaches, the decision-making process of the whole “hybrid” community was similar to that of the “pure” community: the hybrid community—InsBots included—eventually gathered under one of the two shelters. Another result obtained in the study was that, after modifying the InsBots so that they preferred staying in lighter shelters, the whole “hybrid” community occasionally gathered in the lighter shelter: in a sense, the InsBots had proven able to drive the real-life cockroaches towards a decision which was “unnatural” for them. The authors of this study therefore established correlations between the characteristics of a community of robots (P_Rc_) and the behavioural characteristics P_Fc_ of a group of focal living systems. For example, when the robots were modified so that they preferred lighter shelters, the cockroaches gathered in the lighter shelter.^1^ Schematically,

$$ P_{{{\text{Rc}}}} \to P_{{{\text{Fc}}}} . $$

### The structure of research questions in interactive biorobotics

Based on the analysis carried out so far, the structure of the research questions motivating IB studies can be reconstructed in (at least) two ways. On the one hand, IB experiments can be interpreted as addressing questions about what behaviour the focal system will produce when the robot has certain characteristics (or variants of this question adapted to the case of one-to-many, many-to-one, many-to-many interaction). This question can be represented in the following way (note the position of the question mark):

(Q1) P_Ri|c_ → P_Fi|c_?

where the symbol “|” is used as an “or” to formulate a succinct representation of four different questions (e.g. P_Ri|c_ may stand for P_Ri_ or P_Rc_, as in the starling and the crab studies, respectively). Q1 is structurally akin to one of the two “inferential questions” discussed by Gelman (2011) in his analysis of research questions in the social sciences, namely to the “forward causal inference. What might happen if we do X? What are the effects of smoking on health, the effects of schooling on knowledge, the effect of campaigns on election outcomes, and so forth?” (Gelman 2011, p. 955). Thus, for example, the starling study can be read as an attempt to find out where the focal starling will look depending on the fixation point of the robot. The questions motivating the other studies can be easily reconstructed in the same way.

On the other hand, IB studies can be interpreted as attempts to identify what characteristics the robot must have for the focal system to display a certain behaviour (or variants of this question adapted to the case of one-to-many, many-to-one, many-to-many interaction). Schematically,

(Q2) P_Ri|c_? → P_Fi|c_

The starling study can be interpreted as an attempt to find out what behavioural characteristics the robot must display for the focal starling to look at the empty compartment. The questions motivating the other studies can be reconstructed in this way too. Note that Q1 and Q2 refer to proximal studies (see Sect. 2.2), insofar as the antecedent of the implication is a description of the characteristics of the robotic model(s). However, by replacing P_Ri|c_ with P_Ti|c_, one obtains two possible reconstructions of the research questions motivating distal IB studies. Note also that theoretical hypotheses of the form *prox* answer both questions of the form Q1 and Q2. Thus, the starling study, establishing that the focal starling tends to look at the empty compartment whenever the robotic starling does the same, offers a possible answer to a question of form Q1 and of form Q2.

Structures Q1 and Q2 are clearly different from one another. By representing a research question as having structure Q1, one likely assumes that the experimenters will start with a relatively clear idea of the characteristics P_Ri|c_ that the robot(s) will have in the different experimental conditions that will be examined (this does not imply that they do not have an equally clear idea of the possible outcomes in terms of P_Fi|c_). By representing the research question as having structure Q2, one likely assumes that the experimenters start with a relatively clear idea of the possible behavioural characteristics P_Fi|c_ that the focal system(s) may display while interacting with the robot(s), without implying that they do not have an equally clear working hypothesis on the P_Ri|c_ characteristics that the robot(s) must have in the different experimental conditions. Notwithstanding this difference, it will be provisionally assumed here that research questions in IB can be reconstructed both as Q1 and Q2. In the following section, it will be argued that neither Q1 nor Q2 can be properly regarded as requests for explanation; thus, the structural distinction between the two questions will not make the difference as far as the thesis defended here is concerned.

## Interactive biorobotics, explanation, and understanding

### Scientific explanation and understanding

The analysis of the structure of the research questions pursued in IB, and of the theoretical conclusions reached in proximal and distal studies, will be used in this article to reflect on whether IB experiments may advance our understanding of social phenomena in animals by offering explanations of them. To prepare the ground, some remarks on the terms “explanation” and “understanding” are needed. The considerations made in this section are especially meant for readers who are not familiar with the philosophical literature on scientific explanation and understanding. Nothing original will be offered from a philosophical point of view: quite on the contrary, the remarks made here may sound relatively straightforward to philosophers of science. Nevertheless, this section explicitly states some assumptions on scientific explanation and understanding that, albeit philosophically trivial, play a crucial role in the arguments offered in the following sections.

The terms “explanation” and “understanding” are used with several meanings in scientific and common parlance. A teacher explains the Hodgkin-Huxley model to university students, a scientist explains why certain electrical phenomena occur in the cell using the Hodgkin-Huxley model and other assumptions. I can understand the Hodgkin-Huxley model, you can understand why certain electrical phenomena occur in the cell because you can see how they follow from the Hodgkin-Huxley model plus other assumptions. Intuitively, one thing is to explain the content of a scientific model to an audience, another thing is to explain *why* certain phenomena occur(red) using that model. One thing is to understand the content of a scientific model, another thing is to understand *why* certain phenomena occur(red) by realizing that they follow from that model. Scientific research is chiefly concerned with the second sense of these terms: one of the primary goals of science is to *explain why* certain phenomena occur, and science may enable us to *understand why* certain phenomena occur. Scientific research therefore aims at providing explanations, and enables understanding, in a relatively restricted interpretation of the two terms, and it is under this interpretation that the question at issue—whether IB experiments may advance our *understanding* of social phenomena in animals by offering *explanations* of them—is raised here. The ensuing sections will provide reasons to doubt that IB studies conforming to the methodological reconstruction illustrated before end up *explaining why* certain social phenomena occur in animals. One may object that this thesis rests on a restricted interpretation of the term “explain”. Granted, but this interpretation is not ad hoc, crafted only to save the argument: it will be argued that IB studies do not explain social phenomena in the same sense of “to explain” in which scientists are said to explain natural phenomena—where explaining phenomena amounts to explaining why they occur(red).

One of the roles of philosophy of science is to clarify the meaning of general, cross-disciplinary terms used in scientific research. At least from the publication of the seminal article by Hempel and Oppenheim (Hempel and Oppenheim 1948), several analyses of what counts as a “good” *explanation of why* certain phenomena occur(red), and of what *understanding why* certain phenomena occur(red) amounts to, have been offered in the philosophical literature. Providing a comprehensive reconstruction of this research field is out of the scope of this article: the reader is referred to (Salmon 1989) and (Weber et al. 2013) for in-depth analyses. In what follows, some basic considerations on explanation and understanding—in the restricted sense considered here—will be made.

Scientific explanations can be regarded as answers to explanation requests. Explanation requests are typically introduced by “why”—e.g. why, whenever a robotic starling gazes at some point, a real-life starling nearby will tend to gaze at the same point? However, as noted by Bromberger (1966, p. 90), “‘explanation’ may refer to the answers of a huge variety of questions besides why-questions”. A case in point is “how”: a question of the form “how does this system produce this behaviour” may count as a request for explanation, to the extent that clarifying how this system produces this behaviour amounts to explaining why it produces this behaviour. What matters for the present discussion is that explanation requests refer to, or presuppose, phenomena that await explanation. The term *explanandum* (plural: *explananda*) is frequently used to denote the statement expressing the phenomenon to be explained. The question raised above, for example, presupposes the following *explanandum*: whenever a robotic starling gazes at some point, a real-life starling nearby will tend to gaze at the same point.

Two assumptions concerning scientific explanations are commonly (often implicitly) made in the philosophical literature. The first one—relatively basic indeed, and strictly following the considerations made so far—is that, when one tries to explain a phenomenon, that phenomenon (the *explanandum*) must have already been identified: explanatory reasoning starts after encountering—by virtue of serendipity or after some kind of construction or definition process—“some surprising, astonishing phenomena” (Hanson 1958, p. 130). In contemporary analyses of the process of discovery of mechanisms, it is often assumed that the first phase of explanatory reasoning is devoted to the characterization of a puzzling phenomenon to be explained (Darden 2017, p. 258). To be sure, phenomena are often re-characterized (Colaço 2020) also based on the results of previous explanatory attempts. Even in that case, though, there is a logical distinction between the process of explaining a phenomenon and the process of (re)characterizing it. When it comes to explaining a phenomenon, regardless of whether the explanation will lead to a re-characterization of it, the phenomenon itself must have been identified with enough precision.

The second assumption is that the phenomenon to be explained can be an event or a regularity. In the first pages of his *Depth*, Michael Strevens (2008) claims that the objects of explanation can be divided into two classes. “In one class are singular events, such as lights’ being switched on in a particular room at a particular time, and singular states of affairs, such as lights’ being on in a particular room at a particular time” (p. 7). He calls them *events*. “The other class of objects suitable for explanation consists of things that are, each in their own way, robust generalizations”, or *regularities*. The term *phenomenon* is used by Strevens to denote events and regularities when they are objects of explanation (it will be used with this meaning throughout this article).^2^ That scientific explanations have as their object events or regularities is suggested also by Nagel in his *The Structure of Science* (Nagel 1979): “explanations may be offered for individual occurrences, for recurring processes, or for invariable as well as statistical regularities” (p. 15). Bromberger (1966, p. 91) too assumes that the object of explanation can be “an event, a phenomenon, a natural law”.

To sum up. Scientific explanation presupposes the sufficiently precise identification of a phenomenon to be explained, which can be an individual event or a regularity. The statement expressing the phenomenon to be explained is typically called the *explanandum* in the philosophical literature.

Another term of art, *explanans* (plural: *explanantia*), is frequently used to denote what does the job of explaining. Thus, in neuroscience, one can explain why certain electric phenomena occur in neurons of a particular kind (this is the *explanandum*) by showing that they follow from the Hodgkin-Huxley model plus other theoretical assumptions (which form the *explanans*). Scientific explanations can therefore be regarded as structures composed of an *explanandum* and an *explanans*. Not all *explanantia* can satisfactorily explain a given *explanandum*. One of the most important contributions offered by philosophy of science to the analysis of scientific reasoning consists in developing theories on what makes “good” *explanantia* for various kinds of *explananda*. Philosophical models of scientific explanation typically place constraints on the content of the *explanans* (which must cite only theories and facts that are relevant to the occurring of the *explanandum*, according to some notion of explanatory relevance) and on the relationship between the *explanans* and the *explanandum*. For example, the seminal nomologico-deductive model of explanation proposed in (Hempel and Oppenheim 1948) states that the explanandum must *logically follow* from the explanans, meaning that, if the *explanans* had been duly considered in time (by somebody having sufficient logical derivation abilities), it could have served as a basis for predicting the phenomenon described in the *explanandum*. Other models of explanation, which greatly differ from the nomologico-deductive model, can be found in (Salmon 1989; Weber et al. 2013), while more specific discussions about the structure of explanations in the social sciences can be found in (Brady 2011; Coleman 1990; Hedström and Ylikoski 2010; Kincaid 2021; Rudner 1966; Ylikoski 2017).

Note that *testing* an explanation does not involve only testing the particular theories, models, assumptions composing the *explanans*, but also ensuring that the *explanans* provides adequate grounds to explain the *explanandum* phenomenon. For example, suppose that the *explanandum* consists in a certain electric phenomenon occurring in neurons of a particular kind, and that the putative *explanans* consists in the Hodgkin-Huxley model plus other theoretical assumptions. Testing such an explanation does not only involve testing the Hodgkin-Huxley model and the other theoretical assumptions mentioned in the *explanans*, but also ensuring that the “right” explanatory relationship holds between the *explanans* and the *explanandum* (where “right” depends on the model of explanation which is presupposed in the testing process; according to the nomologico-deductive model, for example, one must ensure that the *explanandum* logically follows from the *explanans*).

At this point, a brief remark on the term “hypothesis”, occasionally used in this article, is in order. This term is often used in science to denote newly formulated claims which need (experimental) support. Thus, the claim “whenever a robotic starling gazes at some point, a real-life starling nearby will tend to gaze at the same point” may be called a hypothesis to emphasize the fact that more (experimental) support is needed before it can be accepted by the scientific community. Note that, according to this usage of the term, *in principle* everything is a hypothesis in science: no scientific claim can be rationally said to be totally invulnerable to new evidence or new arguments. Nevertheless, the term is practically useful in scientific parlance to distinguish theses which are still to receive a solid and rigorous evaluation from theses which have already been accepted by a large part of the scientific community. What matters for the present discussion (also to dispel some objections that will be raised in the next section) is that this term, “hypothesis”, is very general and does not denote scientific statements of a particular form or having a particular function in the context of a study. Thus, a regularity such as the starling example may be called a hypothesis to be tested. But the same term can be also used to talk about explanations. Thus, one may say that it is a *hypothesis* that certain electrical phenomena can be explained by reference to the Hodgkin-Huxley model, meaning that one has still to ensure that the “right” explanatory relationship holds between the model and the phenomena to be explained. In this perspective, due to the generality of the term “hypothesis”, testing a hypothesis does not necessarily amount to testing an explanation. Testing the starling hypothesis above, for example, does not amount to testing an explanation because that statement, individually, is not an explanation (for the simple reason that it is a generalization, and not a structure composed of an *explanans* and an *explanandum*).

To conclude, some remarks on the notion of “scientific understanding” is needed. Philosophers of science distinguish between various ways in which this term can be used in connection with scientific reasoning (Barnes 2010; de Regt 2009; Friedman 1974; Hu 2021; Trout 2002). “Understanding a phenomenon” may consists in having a good explanation of why that phenomenon occur(red). On this interpretation—according to which “understanding” and “explanation” are fundamentally synonymous—asking whether IB studies advance our understanding of behavioural phenomena in animals reduces to asking whether IB studies produce good explanations of them. Or, “understanding a phenomenon” may be used to refer to the *psychological* phenomenon which accompanies somebody’s grasping of a good explanation. It is in this sense that, as anticipated in the Introduction, Michael Strevens uses this term: one has a scientific understanding of a phenomenon just in case they grasp a good explanation of that phenomenon (Strevens 2013). According to this interpretation, “understanding” neither is synonymous nor reduces to “explanation”, but one cannot have a genuine understanding of a phenomenon unless a good explanation of it is available. In this perspective, asking whether IB studies advance our understanding of behavioural phenomena in animals involves asking whether IB studies produce good explanations of them.

To sum up. Scientific explanations are structures composed of an *explanans* and an *explanandum*. Testing a scientific hypothesis does not necessarily amount to testing an explanation: some studies contribute to the advancement of science without, per se, producing or testing explanations of phenomena. One understands a phenomenon only if they grasp a good explanation of it. Now recall that, according to several authors, interactive biorobotics may advance our understanding of social phenomena in animals. Building on these considerations, which establish a close connection between understanding and explanation, it is legitimate to ask whether interactive biorobotic studies have explanatory goals and whether they ultimately end up producing or testing scientific explanations of social phenomena in animals. This is the goal of the following section.

### Do interactive biorobotic studies explain biological phenomena?

A *prima facie* reason for doubting that IB has explanatory goals comes from the observation that Q1 and Q2—the research questions addressed in interactive biorobotic studies, according to the analysis made in Sect. 2.3—are introduced by the locution “what”. As such, apparently, they are not questions about why a certain phenomenon is produced: they do not “look like” explanation requests. However, as noted before, explanation requests can be introduced by several locutions. The fact that Q1 and Q2 are introduced by the word “what” is not sufficient a reason to deny that they are explanation requests, and that IB studies have explanatory goals.

Quite on the contrary, one may suggest that Q1 and Q2 are explanation requests, as they both express the intention to identify the determinants of the behaviour of the living system(s) under investigation. With Q1, one asks what focal system(s) reactions will be determined by the robot(s) having certain characteristics. With Q2, one asks what robotic features determine a particular biological reaction. Answers to both questions may assume form *prox* (or *dist*), which express the robotic (or biological) determinants of the behaviour of the focal system(s). Under the assumption that finding what determines, or causes, a given behaviour amounts to explaining it, one may conclude that Q1 and Q2 are explanation requests and that statements of the form *prox* (or *dist*) express causal explanations of the behaviour of the focal system. For example, the goal of the starling study is to find out whether a robotic model of a starling looking at an empty compartment will make a real-life starling look at the empty compartment too. Since this can be interpreted as a question about the existence of a causal nexus between the behaviour of the robot and the behaviour of the focal system, it can be read as a request for a (causal) explanation. Since the study establishes that, when a robotic model of a starling looks at an empty compartment, a real-life starling nearby will tend to look at the empty compartment too, the study may be interpreted as offering a causal explanation of the behaviour of the focal system: the real-life starling looked at the empty compartment *because* the robot displayed a certain behaviour.

This consideration can be attacked from several sides. The thesis that IB studies offer causal explanations of biological behaviours since they establish causal relationship between robotic and biological behaviours rests on the assumption that IB studies establish causal relationships and not mere correlations. This assumption is by no means obvious. However, there is a more straightforward reason to believe that Q1 and Q2 do not express explanation requests, and that neither *prox* nor *dist* offer explanations of biological phenomena. The gist of the argument is that Q1 and Q2 are not explanation requests because they do not presuppose any genuine *explanandum*, i.e. any (description of a) phenomenon awaiting an explanation. Both questions are more properly interpreted as pointing to the definition of a phenomenon to be explained. And the theoretical conclusions supported by IB studies—having one of the forms analysed in Sect. 2.3—do not consist in explanations, i.e. in structures composed of an *explanans* and an *explanandum*. Rather, they consist in regularities linking characteristic of a robot (or a group of robots) to characteristics of a focal system (or a group of focal systems) and awaiting explanation. IB studies thus contribute to the study of social behaviour in animals not because they, per se, offer explanations of social phenomena, but because they define phenomena to be subjected to future research.

This view can be supported using the considerations made in the previous section. As argued before, if the goal of a study is to explain a phenomenon, that phenomenon (the *explanandum*) must have already been identified, and the *explanandum* can be an event or a robust generalization. However, the research questions addressed in IB studies (having form Q1 or Q2) presuppose the identification neither of any well-circumscribed, spatio-temporally located individual event nor of a robust generalization. Consider, for example, the starling study. The goal of the starling study was not to explain why, in a particular moment and space, one particular starling looked at the empty compartment: the study did not aim at explaining any individual event. If this were the case, the study would have started with the description of a particular circumstance in which a starling looked at the empty compartment. The explanation process would have involved identifying, in that particular circumstance, the contextual or internal factors responsible for the event. But the study neither started with, nor pursued, anything like that. Neither the goal of the study was to explain a robust generalization concerning starling gaze behaviour. If it were the case, the study would have started with the description of a generalization stating that, (only) whenever such and such conditions obtain, starlings look at the empty compartment. But the study did not start with anything like that (in fact, it *ended up* establishing such a generalization).

Similar considerations can be made regarding the other three studies. The goal of the bee study was not to explain why, in a particular spatio-temporally circumscribed situation, a particular group of bees approached one food site. Neither it started with a robust generalization, linking the characteristics of the dance to bees’ behaviour, to be explained. The crab and the cockroach studies did not aim at explaining a particular observed event in the life of a crab or a group of cockroaches. Neither they presupposed the definition of a robust generalization stating that, when such and such conditions obtain, crabs choose a mate having particular characteristics, or cockroaches hide under the lighter shelter. Their goal was not to explain already formulated generalizations, but to *define* them. Statements *prox* and *dist* constitute the main theoretical results of IB studies *qua* generalizations to be explained.

In his *Representing and Intervening* (1983), Ian Hacking famously pointed out that [o]ne role of experiments is so neglected that we lack a name for it. I call it the creation of phenomena. Traditionally scientists are said to explain phenomena that they discover in nature. I say that often they create the phenomena which them become the certrepieces of theory (p. 220).

Many scientific experiments, in his view, aim at creating the conditions for some object to manifest some property. In one of his examples, Hall created the phenomenon that bears his name by making a current pass through a gold leaf in a magnetic field: a potential was evoked at right angles to the field and to the current. He uses the term “create” to emphasize that a potential difference with those exact characteristics can be evoked only when somebody creates the right conditions for it, and this may require substantial effort in controlling the world, possibly by building an apparatus. In the creation of a phenomenon, one intervenes in the course of nature to produce stable regularities which, later, can be explained. Not all experiments thus aim at testing explanations of phenomena: some experiments aim at establishing that, whenever some conditions obtain, something happens, i.e. at establishing a stable regularity, a phenomenon (Hacking uses this term to mean “noteworthy discernible regularity”). The phenomenon is created by the scientist, who shapes the world so that it displays the “right” antecedent conditions. Note that claiming that scientists can create phenomena does not imply that any phenomenon can be arbitrarily created: it may be the case that the same initial conditions do not regularly lead to the same consequences, thus, that no stable regularity emerges from a particular way to carve the world. In Hacking’s view, there may be natural phenomena which are virtually non-existent until somebody makes them in the lab, but whether a phenomenon can be created or not, is a question constrained by nature.

IB experiments create phenomena in the sense discussed by Ian Hacking. They involve intervening in the course of nature by building robotic artefacts and creating the conditions for the manifestation of social behaviours that are difficult to observe so clearly “in the wild”, when the focal animal interacts with other animals in uncontrolled environments. By defining new phenomena deserving explanation, IB may greatly contribute to the advancement of research on animal behaviour. Per se, however, IB experiments do not contribute to the explanation or understanding of these phenomena, since their role is to define them.

The considerations made so far can be used to dispel some possible objections to the thesis defended here. As extensively pointed out in Sect. 2.2, it is true that IB studies end up testing biological *hypotheses*. And one may maintain that any experimental study testing a hypothesis, in science, necessarily contributes to the explanation and understanding of some phenomenon: science aims at the explanation and understanding of natural phenomena through the formulation and testing of scientific hypotheses of various kind. Thus—the objection runs—IB studies too contribute to the explanation and understanding of biological phenomena, *contra* what is argued for in this article.^3^ This objection can be dispelled recalling that testing a hypothesis does not necessarily amount to testing an explanation (see Sect. 3.1). Certainly, IB studies contribute *in some way* to the advancement of science: they offer the unique opportunity of systematically manipulating variables (such as cockroaches’ individual preference for lighter shelters, or the gaze direction of starlings) which are difficult to control otherwise. However, they distinctively contribute to science by offering empirical support—through the experimental manipulation of variables which may influence social behaviour—to hypotheses having one of the forms schematized in Sect. 2.2, i.e. to regularities linking characteristics of the robot (or a group of robots) to characteristics of the focal system (or of a group of focal systems). Hypotheses of this form, per se, are not explanations: they are not structures composed of an *explanandum* and an *explanans*. Therefore, IB studies, though testing biological hypotheses, do not test explanations. Note that this conclusion distinctively speaks to the contribution “directly” offered by IB studies to the study of animal behaviour. Per se, as argued here, they do not end up offering explanations. However, they test generalizations that might be used, *in other studies*, for explanatory purposes. For example, the generalization tested in the starling study might be used, in other studies, to explain the behaviour of individual starlings. But the fact that the results of an IB study can be “externally” used in other studies for explanatory purposes does not imply that these results consist in explanations.

Note also that this conclusion concerns IB studies that conform to the methodological reconstruction presented in Sect. 2. They are studies in which the characteristics of a focal system (or a group thereof) are observed after manipulating the characteristics of a robot (or a group thereof). This strategy has been typically adopted in interactive biorobotics so far. However, variants of this methodology, which combine the methodology of interactive and classical biorobotics, can be adopted for explanatory purposes, as it will be suggested in the following, concluding section.

## Concluding remarks: combining classical with interactive biorobotics

It has been argued that interactive biorobotics studies conforming to the methodology typically adopted so far do not lead, per se, to the explanation of social phenomena in animals, but to their definition or creation, in Hacking’s sense. Therefore, under the assumption that understanding a phenomenon amounts to grasping a good explanation of it, they do not contribute to our understanding of social phenomena in animals, *contra* what has been argued by several scholars. Note that this view rests on a particular interpretation of the concepts of “explanation” and “understanding” as they are used in scientific research. However, as stressed before, this interpretation has not been carved ad hoc for the sole purpose of saving the argument. Under this interpretation, explaining phenomena amounts to stating why they occur(red), and understanding phenomena amounts to grasping good explanations of them. It is under this interpretation that explaining phenomena, and advancing our understanding of them, are commonly regarded as important goals of scientific research.

These considerations do not imply, however, that they are the *sole* goals of science: indeed, as pointed out by Hacking, scientific experimentation may also lead to the definition of new phenomena to be explained. Neither this article implies that interactive biorobotics has nothing to offer to the study of animal behaviour. Quite on the contrary, the analysis carried out so far helps us pinpoint the distinctive role that IB can play in behavioural research: robotic models of animals, whose characteristics can be carefully controlled in experimental contexts, constitute unique opportunities for identifying and defining social phenomena to be later understood. By introducing novel *explananda*, which are more difficult to create without the support of robotic technology, interactive robots may really “have the potential to revolutionise the study of social behaviour” (Krause et al. 2011).

It is worth stressing that the analysis carried out here distinctively and specifically concerns interactive biorobotic studies conforming to the methodological reconstruction offered in Sect. 2. Nothing is claimed about other robot-supported methodologies for the study of animal behaviour (in particular, it is not claimed that other robot-supported methodologies cannot lead to the explanation of animal behaviour). And the thesis that IB studies lead to the creation of phenomena, in the sense discussed here, does not imply that this epistemic function is played by IB studies *only*, or that there is anything peculiar about IB as far as phenomena creation is concerned. Other non-interactive robot-supported studies may contribute to the creation of phenomena. Robotic simulations of extinct animals are cases in point (Long 2012; Long et al. 2006; see Tamborini 2021 for an analysis of the role of robotics in the study of animal morphology). Stand-alone robots whose form and control mechanisms supposedly reproduce those of extinct animals may enable one to generate their behaviour, which is clearly unobservable. This strategy has been called *prediction-oriented* in (Datteri and Schiaffonati 2019) and, by reproducing the behaviour of extinct animals, may be regarded as leading to the formulation of behavioural *explananda*.^4^ Interactive biorobotics is only one of the many strategies that may lead to the definition of *explananda* in the study of animal behaviour. These strategies differ from one another in the experimental procedure and, therefore, in the auxiliary methodological assumptions needed to justify the biological relevance of the experimental results. For example, in some cases, one may be led to reflect on the relationship between the phenomena created with the support of a technological apparatus and phenomena that could occur “in the wild”, without any technology-supported intervention. In prediction-oriented, non-interactive studies on extinct animals, one may legitimately reflect on whether the behaviour generated by the robot resembles, to a certain extent, the behaviour that the extinct animal would have generated in the same conditions. Analogously, in *distal* IB studies (see Sect. 2.2), one may reflect on whether the reaction of the focal system to the robot resembles, to some extent, the reaction that it would have generated while interacting with a real-life animal. Different considerations must be made in the two cases, because the methodology of non-interactive, prediction-oriented biorobotics is substantially different from the methodology of distal IB studies. Addressing this issue is out of the scope of this article: see (Datteri 2020a) on the justification of distal IB and (Datteri 2020b) for a methodological reflection on prediction-oriented computer simulation studies that can be extended to the case of robotic simulations. What matters here is that other technology-supported strategies can lead to the creation of phenomena, which may differ from one another in the auxiliary assumptions needed to justify the relevance of the results in connection with the study of animal behaviour.

This said, one may legitimately question whether interactive robots can be used for explanatory purposes too, possibly through variants of the methodology discussed so far. In what follows it will be briefly suggested that the IB approach can be combined with the simulative “synthetic method” characterizing classical biorobotics in a way that may enable one to formulate and test explanations of social phenomena in animals. The strategy sketched here will be tentatively called “simulation-interactive”.

**Fig. 1:**
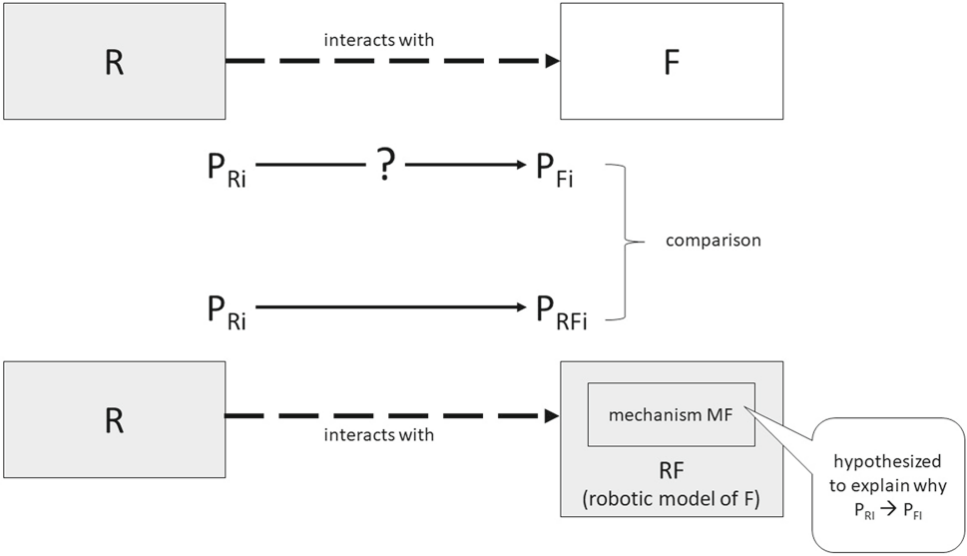
The simulation-interactive methodology, which combines classical and interactive biorobotics for explanatory purposes. R is the robot; F is the focal system. F reacts in a certain way to the robot R (this is the IB part). Why? To address this question, one builds a fully robotic model RF of the focal system (this is the classical biorobotics part) and assesses whether RF reacts to the robot R in the same way as F reacted to R. In that case, one might be led to conclude—with the help of a variety of auxiliary methodological assumptions—that the mechanism MF implemented in the robotic model is responsible for F’s reactions to R, and that it can be used as a basis to explain the phenomenon created in the IB part of the study

Consider a notional, one-to-one IB study that established a regularity of the form P_Ri_ → P_Fi_. Such a regularity states that, whenever robot R displays characteristics P_Ri_, the focal system displays P_Fi_. In our perspective, the authors of this study have created a new phenomenon to be explained. So, at this point, one may legitimately ask *why*, whenever R displays P_Ri_, F displays P_Fi_. According to a well-entrenched and solid perspective, which is widely defended in the contemporary philosophical literature (even though different scholars may disagree on a number of important aspects of it), explaining why living system F regularly displays a certain behaviour in particular conditions—which in our case include the presence of a robot with certain characteristics—requires one to identify the mechanism M_F_ governing the behaviour of the living system and responsible for that particular regularity (see Glennan and Illari 2017 for a comprehensive discussion of mechanistic explanation in various areas of science, biology included). It is at this point that classical biorobotics gets combined with interactive biorobotics. Suppose that one hypothesizes that mechanism M_F_, allegedly instantiated in F, is potentially responsible for the phenomenon created by the IB study. Then, one creates a *robotic model of the focal system*, RF, which implements that mechanism (as it usually happens in classical biorobotics, in which the mechanism to be tested is implemented in a robot). Finally, one establishes an interaction between robot R and robot RF. If the new robot RF, while interacting with robot R displaying characteristics P_Ri_, produces behaviours P_RFi_ which are similar—in the relevant respects and at a certain degree of approximation—to the behaviour P_Fi_ produced by the focal system F in response to the same stimulus P_Ri_, one may be induced to corroborate the hypothesis that M_F_ is the mechanism responsible for the phenomenon P_Ri_ → P_Fi_ (see Fig. 1).

For example, Romano and colleagues (Romano et al. 2020) established one-to-one interactions between locusts of different ages and a robotic model of a lizard. They found out that old locusts reacted (by jumping) *earlier* than young locusts to the appearance of the robotic predator. Moreover, old locusts reacted at a significantly *longer* distance from the robot, compared to young locusts. This is a phenomenon that needs to be explained. The authors sketched a cognitive mechanistic explanation: “the earlier reaction of older adult locusts could be a strategic behaviour (e.g. faster decision-making process), compensating a slower muscular response that may require a longer time to be performed”. They also formulated alternative, neural-level mechanistic explanations that refer to synaptic alterations and sensory threshold in the reactive behaviour of the animals. The IB study does not offer support to any of these possible explanations. One may then proceed combining classical and interactive biorobotics, i.e. implementing a robotic model of a locust (RF) governed by the cognitive mechanism sketched by the author (or by a revised version of it), and assessing whether the robotic locust reacts to the appearance of the robotic predator in a way that is similar, in the relevant respects (time and distance of the first motor reaction), to the reaction of the real-life locust. In that case, one may be induced to corroborate the cognitive explanation offered by the mechanism implemented in RF.

It is not claimed here that this strategy is technically or practically feasible for the explanation of all the phenomena that can be, or have been, created in IB studies. Moreover, it gives rise to a number of methodological issues that will not be discussed here, and that partly overlap with those arising in classical biorobotics. For example, the implementation of a robotic model of the focal system which is able to interact with the “first” robot might require one to include in RF sensory and motor add-ons which are not part of the hypothesis MF under investigation. This methodological problem arises in classical biorobotics too, where the implementation of biological mechanism in a working robot may require one to add electro-mechanical components that are not part of the biological hypothesis and that may introduce unwanted perturbances (for a discussion, see Datteri and Tamburrini 2007; Tamburrini and Datteri 2005; Webb 2006). And, of course, the reproduction of F’s reactions in RF does not offer conclusive reasons to accept MF as an explanation of the phenomenon under investigation: different mechanisms might work equally well. The structure of this strategy and its complexities will be subjected to future studies. This sketchy methodological proposal has been made here to suggest that there may be, “out there”, epistemic uses of interactive biorobots and combinations of classical and interactive biorobotics which have not been thoroughly explored so far, thus corroborating the idea that robotic models may constitute important tools to study social phenomena in animals.

## Data Availability

Not applicable.

## Footnotes

1 Note that robots preferring lighter shelters were able to drive real-life cockroaches *and themselves* under the lighter shelter: the whole hybrid group convened there. Therefore, the study also established a relationship between the characteristics of a group of robots and the behaviour of the same group (i.e. it established that, when the InsBots preferred lighter shelters, the group of InsBots convened with the real-life cockroaches under the lighter shelter). This result will not be considered in this article, which is distinctively concerned with what can be learned on animal behaviour by observing animals’ reactions to robots.

2 Ian Hacking uses the term “phenomenon” to denote a regularity: “a phenomenon is commonly an event or process of a certain type that occurs regularly under definite circumstances” (Hacking 1983, p. 221).

3 The author thanks an anonymous reviewer for this objection.

4 The author thanks an anonymous referee for this suggestion.
